# Association of myocardial fibrosis and prior cardiovascular disease: the multi-ethnic study of atherosclerosis (MESA)

**DOI:** 10.1186/1532-429X-18-S1-O133

**Published:** 2016-01-27

**Authors:** Bharath Ambale Venkatesh, Chia-Ying A Liu, Yuan-Chang Liu, Sirisha Donekal, Ravi Sharma, Yoshiaki Ohyama, Colin O Wu, Wendy S Post, Gregory Hundley, David A Bluemke, Joao A Lima

**Affiliations:** 1 grid.21107.350000000121719311Johns Hopkins University, Baltimore, MD USA; 2 grid.94365.3d0000000122975165Radiology and Radiological Sciences, National Institutes of Health, Bethesda, MD USA; 3 grid.94365.3d0000000122975165Office of Biostatistics, National Institutes of Health, Bethesda, MD USA; 4 grid.241167.70000000121853318Radiology, Wake Forest University, Winston-Salem, NC USA

## Background

Evaluation of myocardial fibrosis has been identified as an important part of studying remodeling processes and arrhythmogenesis. While focal fibrosis associated with tissue necrosis is more likely found in those with prior cardiovascular disease (CVD), whether diffuse fibrosis (associated with cellular apoptosis and other pro-fibrotic processes) is also increased is unknown. We used contrast-enhanced CMR to evaluate the association between CVD in MESA over 10-years with myocardial fibrosis measured at year-10.

## Methods

The MESA study enrolled 6814 participants free of cardiovascular disease at baseline (2000-2002). We included MESA participants who underwent contrast-enhanced CMR at the MESA follow-up exam at year-10 (N = 1840). Coronary heart disease (CHD) was defined as myocardial infarction, resuscitated cardiac arrest, definite angina, or probable angina (if followed by revascularization) that occurred between the baseline and 10-year follow-up exams. We also defined a composite CVD endpoint which included CHD, congestive heart failure, atrial fibrillation, stroke and transient ischemic attack. Using CMR, we characterized left ventricular (LV) myocardial fibrosis with - late-gadolinium enhancement for focal fibrosis (10-12 short-axis and 2 long-axis slices covering the entire LV) and MOLLI T1 mapping for diffuse interstitial fibrosis (1 mid-ventricular short-axis slice). Logistic regression models were used to assess the relationship between the clinical events and the presence/absence of scar by LGE and T1 mapping indices (pre-contrast T1, post contrast T1 at 12 and 25 minutes, and extracellular volume fraction or ECV) after adjustment for demographics - Model 1: age, gender and race; and traditional cardiovascular risk factors - Model 2: Model 1 + blood pressures, hypertension/lipid medication use, diabetes, HDL and total cholesterol, body mass index, smoking status, glomerular filtration rate (post-contrast T1 only). Models with T1 indices were also adjusted for myocardial scar.

## Results

Participants were 68 ± 9 years, 52% male, 45% Caucasian, 9% Chinese, 25% African-American, 21% Hispanic, 55% had hypertension, and 16% diabetes. 146 of 1840 (7.9%) participants were observed to have myocardial scar by LGE. The mean values of T1 indices were - precontrast T1: 977 ± 45 ms; post-contrast T1 at 12': 456 ± 40 ms; post-contrast T1 at 25': 519 ± 41 ms; ECV: 27.1 ± 3.2%. In logistic regression analysis, the presence of LGE scar was strongly associated with prior events (see Table 1). Decreased post-contrast T1 times, indicative of greater diffuse fibrosis were strongly associated with both endpoints. The association between post-contrast T1 and CHD (p < 0.05) remained after adjustment for the presence of myocardial scar by LGE.

**Table 1:** The coefficients (and associated p-values) from logistic regression models assessing the association between prior cardiovascular disease and coronary heart disease with myocardial fibrosis indices.

	Composite Cardiovascular Disease				Coronary Heart Disease			
	No of events/total population	Univariate	Model 1	Model 2	No of events/total population	Univariate	Model 1	Model 2
LGE +ve	143/1840	2.06 (<0.01)	1.64 (<0.01)	1.42 (<0.01)	69/1840	2.53 (<0.01)	2.04 (<0.01)	2.24 (<0.01)
Pre-contrast T1 (ms) × 10^(-3)	108/1344	0.4 (0.87)	-0.7(0.77)	-1.3 (0.59)	55/1344	-3.0 (0.26)	-3.1 (0.30)	-3.7 (0.19)
Post-contrast T1 12' (ms) × 10^(-3)	108/1345	-6.8 (<0.01)	-9.0 (<0.01)	-8.0 (0.01)	55/1345	-8.3 (0.013)	-14.8 (<0.01)	-12.2 (<0.01)
Post-contrast T1 25' (ms) × 10^(-3)	108/1330	-7.5 (<0.01)	-9.0 (<0.01)	-8.2 (0.01)	55/1330	-10.7 (<0.01)	-17.9 (<0.01)	-16.1 (<0.01)
ECV (%)	72/681	0.06 (0.13)	0.05 (0.26)	0.02 (0.58)	36/681	0.04 (0.40)	0.06 (0.29)	0.02 (0.73)

Model 1: age, gender and race; and traditional cardiovascular risk factors - Model 2: Model 1 + blood pressures, hypertension/lipid medication use, diabetes, HDL and total cholesterol, body mass index, smoking status, glomerular filtration rate (post-contrast T1 only).

## Conclusions

Prior CVD events were associated with a greater likelihood of focal LV myocardial fibrosis and a greater amount of diffuse LV interstitial fibrosis in a free-living multi-ethnic population.

